# Diabetes Educators’ Insights Regarding Connecting Mobile Phone– and Wearable Tracker–Collected Self-Monitoring Information to a Nationally-Used Electronic Health Record System for Diabetes Education: Descriptive Qualitative Study

**DOI:** 10.2196/10206

**Published:** 2018-07-26

**Authors:** Jing Wang, Chin-Fun Chu, Chengdong Li, Laura Hayes, Linda Siminerio

**Affiliations:** ^1^ School of Nursing University of Texas Health Science Center at San Antonio San Antonio, TX United States; ^2^ School of Biomedical Informatics University of Texas Health Science Center at Houston Houston, TX United States; ^3^ McGovern Medical School University of Texas Health Science Center at Houston Houston, TX United States; ^4^ School of Medicine University of Pittsburgh Pittsburgh, PA United States

**Keywords:** wearable, connected health, mHealth, diabetes, self-management, lifestyle intervention, electronic health record, self-monitoring, behavior modification, usability

## Abstract

**Background:**

Diabetes educators are integral to a clinical team in providing diabetes self-management education and support; however, current mobile and Web-based self-management tools are not integrated into clinical diabetes care to support diabetes educators’ education efforts.

**Objective:**

The objective of our study was to seek diabetes educators’ insights regarding the development of an interface within the Chronicle Diabetes system, a nationally used electronic health record (EHR) system for diabetes education documentation with behavioral goal-setting functions, to transfer mobile phone- and wearable tracker-collected self-monitoring information from patients to diabetes educators to facilitate behavioral goal monitoring.

**Methods:**

A descriptive qualitative study was conducted to seek educators’ perspectives on usability and interface development preferences in developing a connected system. Educators can use the Chronicle Diabetes system to set behavioral goals with their patients. Individual and group interviews were used to seek educators’ preferences for viewing mobile phone- and wearable tracker-collected information on diet, physical activity, and sleep in the Chronicle Diabetes system using open-ended questions. Interview data were transcribed verbatim and analyzed for common themes.

**Results:**

Five common themes emerged from the discussion. First, educators expressed enthusiasm for and concerns about viewing diet and physical activity data in Chronicle Diabetes system. Second, educators valued viewing detailed dietary macronutrients and activity data; however, they preferred different kinds of details depending on patients’ needs, conditions, and behavioral goals and educators’ training background. Third, all educators liked the integration of mobile phone-collected data into Chronicle Diabetes system and preferably with current EHR systems. Fourth, a need for a health care team and a central EHR system to be formed was realized for educators to share summaries of self-monitoring data with other providers. Fifth, educators desired advanced features for the mobile app and the connected interface that can show self-monitoring data.

**Conclusions:**

Flexibility is needed for educators to track the details of mobile phone- and wearable tracker-collected diet and activity information, and the integration of such data into Chronicle Diabetes and EHR systems is valuable for educators to track patients’ behavioral goals, provide diabetes self-management education and support, and share data with other health care team members to faciliate team-based care in clinical practice.

## Introduction

Research has demonstrated the effectiveness of behavioral lifestyle interventions that focus on goal setting and diet and physical activity self-monitoring to improve glycemic control for patients with type 2 diabetes [1]. Recent studies have used mobile and Web-based technologies to enhance goal-setting and self-monitoring interventions in diabetes care [2,3]. A recent systematic review of the literature on technologies used for diabetes self-management education and support revealed that there were four essential elements in effective technologies that worked on improving glycemic control: two-way communication, analyzed patient-generated health data (PGHD), tailored education, and individualized feedback [4]. However, the highlighted technologies that enabled two-way communication and PGHD did not include the use of electronic health record (EHR) systems that health care provider teams use on a daily basis for care management or for connecting PGHD to EHRs. Furthermore, previous studies have only demonstrated the effective use of mobile [5,6] and Web-based systems [7,8] that were not connected with EHRs in supporting diabetes self-management behaviors. Connected systems that engage both patients and clinical health care providers, including diabetes educators, are essential to ensure expert care for patients with diabetes. It is equally important to also take advantage of mHealth technologies, including mobile phone apps and wearable trackers, to engage patients in self-care behaviors. Thus far, none of the published literature on diabetes education has explored the integration of PGHD collected via mobile technology to EHRs to facilitate the translation of effective technology-based interventions into diabetes education practice.

Diabetes educators are the frontline health care professionals providing diabetes self-management education and support to patients with diabetes across the United States. Diabetes educators can be registered nurses (RNs), registered dieticians (RDs), pharmacists, medical doctors, exercise physiologists, etc [7]. The Chronicle Diabetes system is a nationally used Web-based system for diabetes education documentation with behavioral goal-setting functions, and it is available free of charge to all American Diabetes Association (ADA)-recognized diabetes education programs [9]. Although it is a comprehensive system for diabetes educators to set, document, and track behavioral goals, monitoring of and following up on patient behavioral goals can be challenging for both patients and diabetes educators [9]. To facilitate evidence-based goal-setting and self-monitoring interventions into the diabetes education process, we propose using the Chronicle Diabetes system currently available to diabetes educators to set patient diet and physical activity goals and connecting patient self-monitoring information collected from mobile devices to the Chronicle Diabetes system to facilitate educators’ monitoring of patients’ adherence to their goals. To seek educators’ insights regarding the development of a connected interface within the Chronicle Diabetes system, we conducted individual and group interviews to gain educators’ perspectives on usability and interface preferences in developing such a connected system.

## Methods

### Design

This was a descriptive qualitative study designed to answer the research question “What are diabetes educators’ perspectives on intergrating patient-monitored diet and physical activity into the practice of diabetes self-management education and support?” We conducted semistructured and in-depth interviews to elicit diabetes educators’ views on using an interface built in the Chronicle Diabetes system to connect self-monitoring data collected from mobile devices. To ensure data consistency, one moderator conducted all interviews using a protocol with open-ended questions to obtain diabetes educators’ perceptions on mobile-collected information and their preferences for viewing this information in the Chronicle Diabetes system [10]. The moderators comprised a trained qualitative researcher who understood both the Chronicle Diabetes and Jawbone UP24 systems and another researcher who had developed the Chronicle Diabetes system and was involved in developing the interface in this system to integrate Jawbone-collected information. The study was approved by the Institutional Review Boards of the University of Texas Health Science Center at Houston and the University of Pittsburgh.

### Sample and Setting

We recruited the diabetes educators from western Pennsylvania, where the Chronicle Diabetes system was first implemented, and Houston, TX, where the educators had little knowledge about the system, to get a balanced sample of the educators’ views on the proposed connected interface within the Chronicle Diabetes system. Educators were informed about the study through an email. Diabetes educators who responded to our invitation emails were selected to participate in this study if they were certified diabetes educators practicing in a recognized diabetes education program. When the moderators heard recurring but no new information from the study participants, we stopped the study and did not further recruit study participants. Study participants received a gift card as compensataion for devoting their time to our study.

### Data Collection Procedures

We conducted individual and group interviews in this study. Individual interviews were conducted when study participants could not make it to a focus group session or not enough participants could agree upon a common time for a focus group session. We used a script, including an introductory statement and primary and probe questions, in all of the individual or group interviews. The introductory statement included general statements of focus group to encourage open discussion and diverse opinions; a description of study purpose; and a description of the mobile app, fitness tracker, and Chronicle Diabetes system. We administered a demographic questionnaire to collect diabetes educators’ information. For each primary question, we first asked a general question to prompt the participants’ perceptions and then used “probe” questions to explore any further new information and encourage participants to elaborate on their experience. Our primary interview questions, in particular, were based on the features of the Chronicle Diabetes system interface and the wearable device with its companion mobile phone app. We started our primary questions on how educators use their current EHR system to document diabetes education and support, and then, we asked them to imagine if we had the ability connect mobile device data to their system, how they would want it to look like. We also provided visual aids (ie, PowerPoint slides) of the designs of the Chronicle Diabetes system interface and the mobile phone app to participants and asked them about the features of the “connected” systems that facilitated their education sessions. The interviews were recorded using a digital audio recorder.

### The Electronic Health Record System for Diabetes Education: The Chronicle Diabetes System

The Chronicle Diabetes system provides tools for diabetes educators to document, track, and report on their patients’ education process according to the National Standards for Diabetes Self-Management Education and ADA education recognition program (ERP) requirements. We chose the Chronicle Diabetes system over other EHRs and other Web-based systems in our study mainly due to the following reasons: (1) its detailed features to support goal setting and self-monitoring in diabetes self-management education and support, 2) its potential to be disseminated nationally, (3) its capabilities to connect with various EHRs in the future, and (4) its national impact on supporting diabetes education practice documentation, which will facilitate efforts for securing reimbursement for incorporating digital health solutions in diabetes education practice in the future. Educators use the Chronicle Diabetes system to record patients’ required behavior goals at baseline and continue, modify, or discontinue them at follow-up visits. Behavior goals are categorized as follows: nutrition, activity, medications, monitoring, prevention and treatment of acute complications, prevention and treatment of chronic complications, and psychosocial adjustment or healthy coping. Patient goal achievement at baseline and follow-up can be scored at 0%, 25%, 50%, 75%, and 100%. In addition to goal setting, patient education plans and program processes are recorded. After the one-to-one or group class education session is completed and documented, all aspects of the education process are automatimatically summarized in a format that meets ADA ERP documentation requirements.

### Patient-Monitored Diet and Activity Data Using UP24 Jawbone Wristband and Companion Mobile Phone App

UP24 by Jawbone is a wristband that objectively tracks individuals’ physical activity and sleep; its companion mobile phone app shows this tracked information and includes features to search or track food intake. We compared a few commercially available fitness trackers prior to the study and chose the Jawbone UP24 in this study due to its feature of distingushing between calories burned from planned exercise minutes and resting calorie burn. This feature supports the evidence-based self-monitoring intervention used in the Diabetes Prevention Program that focused on tipping the calorie balance through noting the calories burned through planned exercise. It should be noted that this device is no longer in production or actively supported. However, the knowledge gained from using UP24 by Jawbone can be transferred to other fitness tracker products that are now providing the same features as this device.

### Data Management and Analysis

All audiotapes were transcribed verbatim in English by a professional transcription service company. Any information that could refer to patient data in the transcripts was either removed or de-idnetified. Two trained researchers analyzed the transcribed data and the notes taken during the interviews using conventional content analysis [11,12] for thematic patterns based on the categroies derived from coded text segments. Concepts or emerging thematic patterns were frequently cross-checked within and across transcripts to ensure data quality. The interpretation of the data was reviewed and refind by the investigators; two coders had open discussions to achieve agreement when there were discrepencies in data interpretation. Common themes related to the educators’ experience in using the Chronicle Diabetes system interface and wearable devices and example quotations are presented below in the Results section.

## Results

### Sample Characteristics

We recruited 8 diabetes educators (3 RNs and 5 RDs) from Pittsburgh, PA, and Houston, TX. They had an average of 22 years of general practice experience and an average of 13 years of practice experience in diabetes education. On average, the participants had approximately 1.75 years of experience using the Chronicle Diabetes system.

### Thematic Analysis Findings

Five individual interviews and one focus group session were conducted. Each interview or focus group session lasted from 30 to 60 minutes, and all six sessions generated a total of about 233 pages of transcribed text. The five common themes that emerged from the interview responses are described in the following subsections, which also include excerpts from the interviews or focus group sessions.

#### Enthusiasm and Concerns About Viewing Self-Monitoring Data in the Chronicle Diabetes System

All diabetes educators expressed strong enthusiasm toward viewing diet and physical activity information in the Chronicle Diabetes system. Some educators were more enthusiastic about certain diet and activity data than others. Most educators were not very enthusiatic about seeing sleep data. They did not think monitoring sleep was necessary, unless patients had sleep disorders; others felt that sleep data are beyond the scope of diabetes education practice, while one educator thought that knowing patients’ total hours of sleep may be helpful for understanding their general health.

If I was visually looking at it, my number one things would be calories, carbs, protein, and fiber...But I work in the Weight Management Center, and protein and fiber are all we really focus on.

Total active time...total burn calories would be important for me...I would like to know total times that they’re active throughout the day...total burned calories would be important so we know...their caloric intake is matching up or negative, if they want to lose weight.

I wouldn’t exactly know what to do with all the information (sleep). Like, if some, if somebody...sleep apnea, you could talk about it, but it’s not something I’m as familiar with as a diabetes educator.

Some educators expressed concerns regarding how often patients would monitor their activity. They thought that constant self-monitoring might cause patients to feel overwhelmed. Instead, they preferred that patients monitor themselves for only a few weekdays or only on weekends, whereby educators could still detect discernable patterns in patients’ self-monitoring behaviors. The educators also expressed concerns about patient compliance. They questioned whether data collected on mobile devices would exactly reflect patients’ activities because some patients may log their data based on how they would like their providers or educators to view their behaviors. These may impact their decisions when they view the connected self-monitoring data during a diabetes education visit.

Don’t overload them.

Don’t put the expectations so high.

So there’s a lot of information on that page that might be a little bit overwhelming, even from my perspective, as a professional.

It would be used mainly for educational reasons, but after a while...I think that patients would get wise to it and put in the foods that look good...

After a couple of months, if they’re seeing that they’re not pleasing their physician or whosever is looking at this stuff, they’ll start putting in the things that they know calculate to be the right thing.

#### Varied Preference Toward the Kinds of Details Displayed Depending on Patients’ Needs, Conditions, and Behavioral Goals and Educators’ Training Background

The educators valued viewing detailed dietary macronutrient and activity data from patient self-monitoring. When viewing patient self-monitoing data, some educators factored in patients’ health conditions and status to select specific information that would be more relevant to individualized diabetes education.

RDs for sure want to track food logs and carb counting, fiber, and calories...

Fiber, protein, calories are important for weight loss purposes...Depending on the patient issues, the needed values are different...personalize which factors to see individualized to each patient. For example, if the patient has high lipids, cholesterol is important to see. If the patient has kidney issues, protein and sodium are important to see.

Some educators preferred viewing information about calorie counting and carbohydrates, whereas others wanted to see percentages of calories from fat, carbohydrates, and proteins in a graphical chart on a daily basis.

Most say macronutrients are more important than percentages, while RDs may have different views. Some want to calculate carbs specifically from sugar and calculate other carbs altogether...Others want to see what percent of carbs come from sugar.

My number one things would be calories, carbs, protein, and fiber...But I work in the Weight Management Center, and protein and fiber are, like, all we really focus on

#### Preference for a Diabetes Education System That is Connected to and Integrated Into the Electronic Health Record

All the participants found integrating mobile phone- and wearable tracker-collected self-monitoring data into the Chronicle Diabetes system extremely helpful and particularily favored integrating the current EHR system with the Chronicle Diabetes system. Documenting detailed diabetes self-management education and support information, along with required information for education program recognition in the Chronicle Diabetes system and required documentation in the hospital or practice EHR system, should be all integrated into one connected system.

Then we wouldn’t have to double document it. If we could put it in there and it would automatically go, that would be nice.

Well that’d be great because then I wouldn’t have to chart so dang much. Like, it—all the information would already be in the chart.

#### Interprofessional Team-Based Care Using Connected Electronic Health Record

A health care team and a central EHR system need to be formed for educators to share summaries of self-monitoring diet and physical activity data to communicate with other health care providers in diabetes education. Most educators agreed that the patient-monitored diet and activity information would be helpful for primary care providers, such as family physicians. Some said that they would like to see all self-monitoring data, but other educators thought that physicians would not have time to use the Chronicle Diabetes system. Instead, they thought that physicians may be able to obtain information indirectly from other health care providers, such as an educator or a dietician. The diabetes educators wanted a space in the Chronicle Diabetes system to include comments and share opinions with other health care providers. The preferences regarding the frequency and timing of comments varied across the educators.

The information in Chronicles will be good supporting data.

They would develop teams. It would be the educator on the team. If there was a team approaching a physician practice...to collaborate and take [it] to the physicians...that would be very helpful. It would have to come from another health professional...

And you would think that they would look at using this kind of technology, but they’re being pushed in so many different directions...I’m sure they don’t perceive that they have the time for this.

I don’t know if people have time to read. I think doctors like to have it there and if they need to refer to it, they really like that.

Primary care physicians or the nurse practitioners, physician assistants (should be able to view the data). Whoever their primary care is and whoever is following them through the diabetes (treatment).

No.

I wish we could say yes.

They don’t. Unless—they would develop teams. It would be the educator on the team. If there was a team approaching a physician practice

If Rob had this data to collaborate and take to the physicians...Yes...that would be very helpful. It would have to come from another health professional...Right...to the physician to pay attention to it. The patient brought it in. They’d scan through it. Say—pretend like they were really digesting it and say, “Oh. This is great! You need to exercise more.”

#### Advanced Features for the Mobile App, Chronicle Diabetes system, and the Display of Patient Self-Monitoring Data

For the mobile app, educators suggested adding voice search rather than barcode scanning or manual entry to help patients log their foods more easily, as well as a function that automatically records physical activity to allow more accurate real-time data.

If they had an app that could—if you could...talk into your phone...and it types what you say...People would love that as opposed to choosing it on an app where you have to go in or the one that reads the scanning bars.

Some educators suggested adding a new function to Chronicle Diabetes system that would allow them to merge their preferred self-monitoring data at once to observe the effects of multiple behavior interactions in diet and activity with blood sugar or weight.

When we’re talking about blood glucose...in addition to just knowing the number or the time of day...if they’re exercising, you can track their blood sugar level in relation to movement...And the same with food...

I need a place where the patient can put in their carbs, their weight, their calories, and their glucose all in one place and to be able to see how the amount of carbs that they ate corresponded to their blood sugar...

I would like the blood glucose to interact with the exercise and the blood glucose to interact with the nutrition.

Several educators wanted to view a variety of data (eg, nutrition, exercise, blood glucose) in separate tabs in Chronicle Diabetes system and suggested different formats for daily, weekly, or monthly summary reports.

The blood sugar level, and on the graph, show the exercise...but I know I would want a separate tab for exercise and a separate tab for nutrition.

I would like to have a tab that—where you can see all the blood sugar levels no matter what time of the day.

I would want to look at the carbs and the protein and...I would want to look at their total fat intake as well...

I like the summary ones the best, for the week...I’m not liking the daily one again as much...

If they’re coming to me with a problem, I want to look at the past seven days more than the month because then it’ll dilute the statistics...

## Discussion

### Principal Findings

Our study demonstrates that diabetes educators are enthusiastic about the incorporation of self-monitoring information into the Chronicle Diabetes system to monitor patients and ensure patient adherence to behavioral goals during diabetes self-management education and support. Integrating such data into a centralized system facilitates robust data collection, synthesis, and analysis and has the potential for developing precision diabetes management with context-aware, individualized guidance presented to the patient and caregivers in a coordinated fashion [13]. The diabetes educators’ insights regarding information display, interface design, and data integration effectively show how the wearable fitness tracker and its companion dietary self-monitoring app can support diabetes educators’ clinical work to improve diabetes self-care behaviors, care coordination, and patient outcomes. The 2017 National Standards for Diabetes Self-Management Education and Support recommend the provision of individualized diabetes education based on the patient’s medical history, health beliefs and attitudes, diabetes knowledge, and self-management skills and also support the use of evidence-based technology–based solutions for delivery of diabetes self-management education and support [7]. A recent study in the United Kingdom exploring patients’ unmet diabetes self-management education and support needs highlighted their needs for support in behavior change, particularly in physical activity and dietary changes, using digital technology [14]. Our findings indicate that diabetes educators prefer the reporting of self-monitoring information that is closely relevant to improving diabetes outcomes and is within the scope of diabetes educators’ practice. Accordingly, patients’ diet and physical activity reports were thought to be of greater relevance than sleep activity since the latter was deemed to be beyond the scope of diabetes education practice. Future studies can explore whether summarizing sleep data with actionable insights would address educators’ lack of confidence in handling sleep data. The educators in our study also suggested that all types of macronutrient (eg, protein, carbohydrates, calories, fat) and activity (eg, exercise type and duration, steps) information are needed so that educators with different training backgrounds can always find information relevant to enhance diabetes education and outcomes, even for patients with different health conditions.

While the mobile app itself does not provide personalized education or therapeutic support to patients, the integration of mobile-based self-monitoring information into the Chronicle Diabetes system would overcome this limitation by enabling educators to focus and interpret self-monitored diet and exercise information that is relevant to specific patient needs, rather than spending education time on dietary recalls with patients. Other studies have recommended employing a health coach or a certified diabetes educator who is not part of a health care team to provide diabetes self-management support based on mobile phone- or wearable tracker-collected self-monitoring data [2,8]. Our study assessing insights from diabetes educators who are part of a current health care team can provide some unique perspectives. The 2017 National Standards for Diabetes Self-Management Education and Support recommend the use of evidence-based technology-based solutions for delivery of diabetes self-management education and support [7].The findings from our study not only expressed educators’ insights regarding using mobile- and wearable tracker-collected self-monitoring data in one diabetes education EHR system but also provided foundational knowledge in facilitating other evidence-based digital health solutions to be integrated into clinical workflow through EHR integration. With the increasing use of digital health and wearable solutions, there is expanding availability of PGHD to diabetes educators. The advantages of utilizing such data are obvious; even no data or missing data could provide meaningful information regarding patients’ engagement with their self-management and lead to product patient-educator conversations. However, potential shortcomings deserve further research and attention. The usability of connected digital health solutions need to be firmly addressed to provide summarized PGHD with actionable and relevant information to facilitate current clinical workflow, and it should not take clinicians away from their interaction with patients. Our educators also expressed concerns regarding patient compliance and data accuracy, and other matters (like patient privacy and confidentiality, data ownership, information overload, liabilities, efficiency and clarity of the data, lack of reimbursement) are also potential issues that deserve further research.

In this study, diabetes educators expressed the need to connect the Chronicle Diabetes system with existing EHR systems for managing patient care and expressed a desire to play a more active role in reviewing mobile data and connecting with physicians and other prescribing providers such as nurse practitioners to provide team-based care. In a previous study in which patients were provided mobile self-management support, the physicians’ prescribing behaviors did not seem to change [15]. Additional research is needed to determine whether an integrated system with mobile data connected to the EHR system would influence physicians’ prescribing behavior through an interprofessional team-based approach, where a diabetes educator takes the leading role to review the summarized PGHD.

### Limitations

There are several limitations to this study. First, our study sample was small; thus, it may not be representative of the general diabetes educator population. Second, there may be self-selection bias in our sample because all diabetes educators were self-selected into the study after seeing the advertisement. Third, we only chose one mobile app with patient-monitored diet and activity information to show to the diabetes educators at the interviews; viewing other brands with different interfaces may trigger different insights from the diabetes educators. Future research is needed to expand the sample size and representation and to include other PGHD pertinent to diabetes self-management education and support practice.

### Conclusions

A full range of tracking details of mobile phone- and wearable tracker-collected diet and activity information is needed to support educators’ preferences, and the integration of such data into Chronicle Diabetes and EHR systems is valuable for educators to track patients’ behavioral goals and provide precise diabetes self-mangaement education and support. Diabetes educators’ perspectives need to be incorporated as we develop future mobile and connected systems to support team-based diabetes care and education in clinical practice. The study findings were used to inform the development of a connected interface in the Chronicle Diabetes system to integrate Jawbone-collected self-monitoring diet and physical activity information, and the connected system is currently being tested in a multisite randomized clinical trial [16].

## Abbreviations

ADA: American Diabetes Association
EHR: electronic health record
ERP: education recognition program
PGHD: patient-generated health data
RD: registered dieticians
RN: registered nurses
